# Supplementary material for: Prevalence and associated factors of delayed first antenatal care booking among reproductive age women in Ethiopia; a multilevel analysis of EDHS 2016 data
Source: PLoS One. 2020 Jul 6;15(7):e0235538. doi: 10.1371/journal.pone.0235538 (PMC7337309; doi:10.1371/journal.pone.0235538)
Supplement: Attachment — Submitted filename: Comments - 1.docx [file pone.0235538.s001.docx]

Lines 107-109. The author refers to the Ethiopian Census 2007 as the sampling frame of the EDHS 2016. This may not be very correct as DHS surveys only use the Census data to select Enumeration Areas. Then, a listing exercise is conducted in each selected EA to update the list of households. The updated list of hhds is used to select households to interview. The authors could revise the statement according to this methodology.

A conceptual framework to help readers understand why these variables were included in the study should have supported the description of the study variables

Lines 145-147. Sample weights should be used throughout the analyses, not only for frequencies and for proportions. Could the author clarify whether the multilevel analysis was weighted or not.

Were collinearity checks performed on the independent variables before ruining MV analyses?

The paragraph on the random effects does not add value to the paper; it rather brings statistical information that the author seems struggling to explain properly. The author uses values (va, π) that are not documented in the paper. Furthermore, I would expect the author to first clarify why a random effect model was needed on these data and then explain what those effects (if any) could be referring to in practice.

Presenting the results section by random vs. fixed effects seems to turn the paper to a methodological paper, comparing methods, rather than keeping the focus on describing the factors associated with delayed ANC. In addition, a number of statements in this section are subjective interpretations of statistical models which are not the focus of the paper and not expected in a result section. This section should be entirely rewritten to clearly highly the key results of the analysis that would be discussed later.
