# Supplementary material for: Prevalence and associated factors of delayed first antenatal care booking among reproductive age women in Ethiopia; a multilevel analysis of EDHS 2016 data
Source: PLoS One. 2020 Jul 6;15(7):e0235538. doi: 10.1371/journal.pone.0235538 (PMC7337309; doi:10.1371/journal.pone.0235538)
Supplement: Attachment — Submitted filename: Response to reviewrs.docx [file pone.0235538.s003.docx]

**June 13, 2020**

**Response to editor/reviewers**

**Title:** Prevalence and Associated Factors of Delayed First Antenatal Care Booking among Reproductive Age Women in Ethiopia; A multilevel analysis of EDHS 2016 data

Manuscript number: PONE-D-19-33776R1

**Editors comment**

We noticed you have not corrected several occurrences of overlapping text with previous publications, which needs to be addressed.

In your revision ensure you cite all your sources (including your own works), and quote or rephrase any duplicated text outside the Methods section. Further consideration is dependent on these concerns being addressed.

**Authors response**: Dear editor thank you for your concern. We have checked our paper/manuscript in advance and we corrected text overlaps.
